# Radiographic Accuracy and Consistency of Implant Placement Using the ROSA^®^ Knee System in Robotic-Assisted Total Knee Arthroplasty: A Prospective Analysis

**DOI:** 10.3390/life16060925

**Published:** 2026-06-01

**Authors:** Wai Kit Wong, Naim Che-Kamaruddin, Siti Zubaidah Zulkhairi, Shi Yao Chong, Abdullah Suhail, Suresh V Nainan George, Teong Wan Ewe, Hwa Sen Chua

**Affiliations:** 1 Orthopaedic Centre of Excellence, Sunway Medical Centre, Bandar Sunway, Subang Jaya 47500, Selangor, Malaysia; 2 Clinical Research Centre, Sunway Medical Centre, Bandar Sunway, Subang Jaya 47500, Selangor, Malaysia; naimchekamaruddin@gmail.com (N.C.-K.);; 3 Sunway Medical Centre, Bandar Sunway, Subang Jaya 47500, Selangor, Malaysia; chongshiyao@gmail.com

**Keywords:** robotic-assisted total knee arthroplasty, ROSA, rotational accuracy, collaborative robot, personalized alignment strategies

## Abstract

The integration of robotic systems in knee arthroplasty has accelerated, with early results indicating improved precision and outcomes. The ROSA^®^ Knee System facilitates the robotic-assisted positioning of cutting guides while enabling real-time assessment of ligament balance, with the surgeon retaining control of all bone resections. This study aimed to evaluate the precision of implant positioning, achievement of the planned hip–knee–ankle (HKA) axis, and consistency across different operating surgeons. A prospective observational study was conducted on patients undergoing robotic-assisted total knee arthroplasty (RATKA) using the ROSA Knee System. Radiographic assessments included pre- and post-operative AP/lateral knee and long-limb radiographs, supplemented with post-operative CT to assess femoral component rotation. Intra-operative alignment data recorded by the ROSA console were compared to post-operative imaging. Complication rates were also evaluated. Among 94 knees analyzed, the mean difference between intra-operative and post-operative HKA measurements was 1.66 ± 2.09°, which was statistically significant. Minor but significant differences were observed in LDFA (0.32 ± 1.84°, *p* = 0.034), MPTA (0.84 ± 1.17°, *p* < 0.001), and femoral component rotation (1.63 ± 2.40°, *p* < 0.001). No significant variation was noted in LDFA or MPTA between surgeons, though differences were present in rotational alignment and HKA. The intra-operative HKA showed moderate correlation with both measured post-op HKA and arithmetic HKA (aHKA), with slightly stronger correlation with the latter. The ROSA Knee System enables reproducible and accurate implant positioning with reliable restoration of the planned HKA axis. Once a surgeon is proficient, results are consistent without increased complication risks.

## 1. Introduction

Despite advances in total knee arthroplasty (TKA), a subset of patients continues to report dissatisfaction following surgery. Historical data suggested satisfaction rates of approximately 80% [1,2], though more recent figures indicate improvement closer to 90% [3]. While patient satisfaction is multifactorial, the precision of component placement and meticulous handling of peri-articular soft tissues remain pivotal in optimizing outcomes [1,4,5]. The development of robotic technology and its rapid adoption in arthroplasty surgery aim to ameliorate these, with a goal of improving overall outcomes and satisfaction.

Robotic platforms are generally classified as fully active, semi-active, or passive [6,7]. Fully active systems function autonomously to execute bone resections. Semi-active robotic units offer real-time intra-operative feedback about implant positioning, gap balance and overall alignment; however, the surgeon retains full control and performs the actual bone cuts. Passive systems are essentially navigation systems that provide detailed anatomical data and recommendations for bone resection and implant placement without direct control over instrumentation. Fully active systems were at the forefront during early adoption of robotic systems, but due to the increased complication rates and technical challenges, their initial adoption was not sustained [6]. Semi-active systems came to prominence with the introduction of the Mako SmartRobotics^TM^ System (Stryker US (Mako Surgical Corp), Fort Lauderdale, FL, USA). Since then, numerous other robotic systems have been introduced, and their adoption has been rapid due to various benefits, such as more accurate implant placement, more objective gap balancing, reduced rates of inadvertent peri-articular soft tissue injury, and good early outcomes [5,8,9,10,11,12,13].

The ROSA Knee System (Zimmer CAS, Montreal, QC, Canada) is a relatively new system that involves the robotic placement of the cutting guide and dynamic real-time assessment of ligament balance [6,7,14,15]. It has been described as a “collaborative robot” or “co-bot”, where the robot aids in placement of the cutting jig but leaves bone resection to the surgeon, mirroring conventional techniques [6,12,16]. Data regarding the accuracy of this robotic system remains inconclusive [17,18]. The primary aim of this study is to assess the system’s ability to achieve precise implant alignment and obtainment of the desired hip–knee–ankle (HKA) axis. Secondary objectives include determining inter-surgeon consistency and evaluating how the robot-captured intraoperative HKA compares to both post-operative and arithmetic HKA (aHKA). This study is purely an assessment of radiological outcomes, with the assessment of clinical function being planned out for future research. Our hypothesis posits that the ROSA Knee System will be able to offer consistent attainment of the desired HKA axis as well as accurate and precise implant placement, demonstrate reproducible alignment outcomes among different surgeons, and that there will be good correlation between the robot-captured HKA with both the measured post-op HKA and aHKA.

## 2. Materials and Methods

### 2.1. Patient Selection

This is a prospective observational study conducted after obtaining ethical clearance from Sunway Medical Centre Independent Research Ethics Committee (SREC No.: 020/2022/IND/ER). All consecutive eligible patients meeting the inclusion and exclusion criteria as outlined in Table 1 were offered participation in this study and agreeable patients enrolled. Written informed consent was obtained from all patients participating in this study.

Pre-operative data collected included age, gender, weight (kg), height (m), body mass index (BMI), and laterality. We also collected data on intra-operative and early post-operative complications, such as tracker pin site fractures and infections, wound dehiscence, surgical site infections, thromboembolic events, as well as implant notching and overhang.

### 2.2. Surgical Technique

The ROSA Knee System is a collaborative robot that offers the surgeon an option to proceed with operative planning using either an image-based method, which requires specific two-dimensional (2D) radiographs to generate a virtual 3D model, or an imageless model where there is a reliance on the intra-operative determination of bony landmarks and evaluation of ligament balance. Detailed surgical technique has previously been extensively published [7,16,19].

All surgeries were performed via a medial parapatellar approach using cemented minimally constrained knee implants (Cruciate Retaining, CR or Posterior Stabilized, PS; Persona^®^ Knee System, Zimmer Biomet, Warsaw, IN, USA) without patellar resurfacing. The cases included in this study were performed using the image-based workflow.

After necessary exposure, the robot was calibrated via registration of bony landmarks. Baseline deformity and range of motion were then determined. Adopting personalized alignment strategies, the surgeon then adjusted and confirmed the planned resections to allow obtainment of the desired implant position and alignment. After surgeon confirmation, the ROSA Robotic Arm positioned the cutting jig in the correct position. At this stage, the femoral rotation was assessed using the ROSA Femoral Rotation Tool, which incorporates the FuZion^®^ system (Zimmer, Warsaw, IN, USA) to assess and balance both the extension and flexion gaps. Once that was determined, the cutting jig’s position was secured via pins, and the surgeon completed the bone resections using an oscillating saw as per convention. The remainder of the TKA procedure from trial implant assessment, irrigation, cementation, up until closure was as per conventional TKA. The overall flow of surgery would be more in line with the surgeon’s operative habits and muscle memory; however, it must be pointed out that ROSA Knee does not aid in the actual resection through haptic limitations of blade, burr or drill placement.

All four surgeons who participated in this study are highly experienced robotic arthroplasty surgeons at a high-volume robotic arthroplasty centre with an annual case load exceeding 800 joints. Specific to the ROSA system, each of them had completed training and certification together prior to the official launch at our institution and have long overcome the challenges associated with the learning curve of adoption. Each surgeon has gone on to log more than 50 cases before the commencement of this study.

### 2.3. Radiographic Protocol

All patients were subjected to a specified protocol of long-limb radiographs, along with an AP and lateral view of the knee. The same set of radiographs were then repeated at 6 weeks post-TKA, together with a CT scan of the knee to permit assessment of the accuracy of the rotational alignment of the femoral component. The radiological images were stored on a PACS system, and measurements done using the DICOM viewer measurement tools by blinded evaluators.

The hip–knee–ankle (HKA) axis is defined by the angle subtended by the mechanical axes of the femur and tibia. The mechanical axis of the femur is represented by a line connecting the centre of the femoral head and centre of knee, and the tibial mechanical axis is represented by a line between the centre of the knee and centre of the ankle. The centre of the femoral head is determined using the concentric circle method, and the centre of the ankle is represented by the midpoint of the talus [20].

The lateral distal femoral angle (LDFA) was defined as the lateral angle subtended by the mechanical axis of the femur and the distal femur joint line. The medial proximal tibial angle (MPTA) is defined as the medial angle between the mechanical axis of the tibia and the proximal tibial joint line [21].

**Figure 1:**
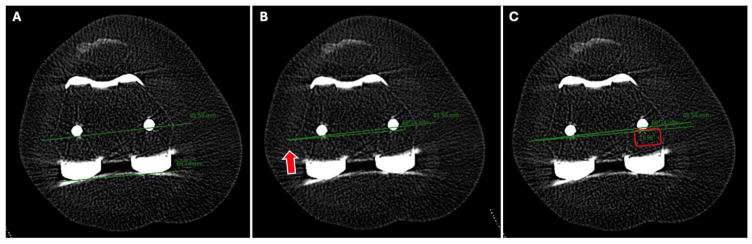
Measurement of femoral component rotation on post-operative axial CT scans. (**A**): One line represents the trans-epicondylar axis (TEA) and another line is drawn connecting the most posterior aspects of the femoral component (posterior condyle line). (**B**): The posterior condyle line is transposed (red arrow) to the TEA. (**C**): The angle subtended by both these lines is taken as the femoral component rotation and is highlighted here in the red box.

For the assessment of rotational accuracy, femoral component placement was measured on axial slices of the CT scan and is represented by the angle subtended by a line representing the trans-epicondylar line (TEA) and another line connecting the most posterior aspects of the femoral component (posterior condyle line). This is in keeping with the publications of Berger et al. [22] and Adamska et al. [23], and is better depicted in Figure 1 below.

**Figure 2:**
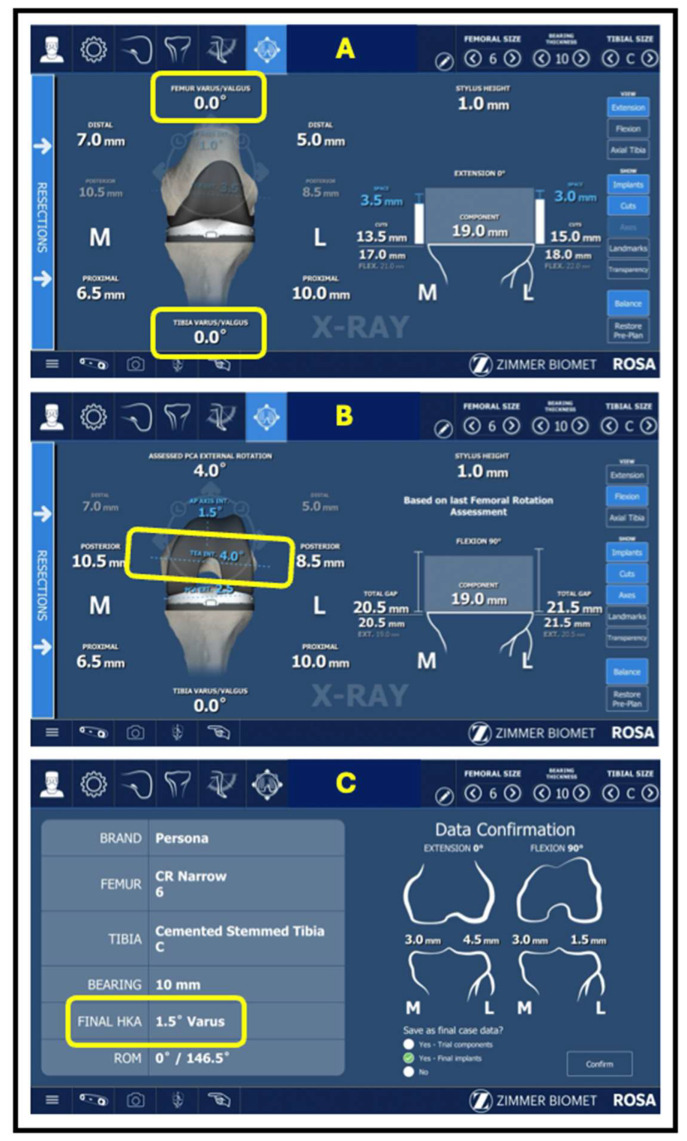
Example of patient data presented by ROSA Knee software intra-operatively. (**A**): Varus/valgus position of the femoral and tibial components highlighted. These values are used to calculate the LDFA and MPTA. (**B**): Femoral component external rotation visualized on this planning page intra-operatively. (**C**): Final HKA obtained is shown here and is used for comparison against the measured post-op HKA and aHKA.

These post-op measurements were then compared to intra-operative readings extracted from the robotic console, as shown in Figure 2 below.

### 2.4. Data Analysis

A post hoc power analysis was conducted using the observed primary outcome (difference between intra-operative and post-operative HKA measurements). Power was estimated using paired effect sizes (Cohen’s d_z_) with α = 0.05.

Descriptive statistics were used to summarize demographic variables, including mean, median, standard deviation, range for continuous data (age, weight, height, BMI), and frequencies and percentages for categorical data (gender, ethnicity, laterality). The distribution of continuous variables was assessed for normality using the Shapiro–Wilk test prior to inferential analysis. As the variables did not meet assumptions of normality, non-parametric methods were applied. Non-parametric Kruskal–Wallis tests were used to assess differences in alignment parameters (Hip–Knee–Ankle axis [HKA], Lateral Distal Femoral Angle [LDFA], Medial Proximal Tibial Angle [MPTA], and Rotational Alignment [ROT]) between surgeons and between post-operative and intra-operative measurements. As the evaluated alignment parameters represented predefined study outcomes of clinical interest, adjustments for multiple comparisons were not applied, and findings should be interpreted within an exploratory framework.

Intra- and inter-observer reliability was analyzed using Intraclass Correlation Coefficients (ICC) from a subgroup of 30 knees measured by three independent raters. The measurements were repeated at one-week intervals to complete three sets of readings. All analyses and data visualization were conducted using R statistical software (version 2023.12.0+369). Results are presented with 95% confidence intervals where relevant to aid interpretation of the magnitude and precision of observed differences. A *p*-value of less than 0.05 denoted statistical significance.

All investigators and procedures undertaken were in accordance with the ethical standards of the institutional research committee and with the 1964 Declaration of Helsinki and its later amendments or comparable ethical standards.

## 3. Results

At our centre, 338 RATKAs using the ROSA Knee System were performed from December 2022 until December 2024 by four senior board-certified arthroplasty surgeons. A total of 210 patients had to be excluded as they either did not meet the inclusion and exclusion criteria or were not agreeable for study enrollment. A total of 128 patients were enrolled in the study. Out of those who were enrolled, 34 patients had to be excluded as they did not complete their post-operative radiographic assessments and were not agreeable to return to complete these scans. A final tally of 94 knees was available for final analysis and formed the basis on which this study was conducted. A post hoc power analysis based on the primary outcome (difference between intra-operative and post-operative HKA measurements) demonstrated that this sample size provided >99% power to detect meaningful differences at α = 0.05.

### 3.1. Patient Demographics and Cohort Characteristics

All 94 patients suffered from end-stage bi- or tri-compartmental knee OA and had exhausted all attempts at conservative treatment. The cohort consisted of 62 women (66%) and 32 men (34%), with a mean age of 66 years (range 45–81). There were 48 right knees (51%) and 46 left knees (49%). Average follow-up duration was 7.15 months (range 3–24 months). Mean BMI was 28.6 kg/m^2^ with 60.6% of patients being in the pre-obese and class I obesity categories. Surgery duration averaged 83.6 ± 23.9 min.

### 3.2. Alignment

The average pre-op HKA axis was 6.7 ± 8.8 degrees varus and this improved to 3.8 ± 4.4 degrees varus post-operatively. Comparing the post-op measurements against verified intra-op values, there was a mean difference of 1.66 ± 2.09 degrees, which was statistically significant. This is clearly depicted in Table 2 below.

### 3.3. Accuracy of Implant Positioning

Comparison of post-operative measurements against the verified intra-operative implant positions yielded a statistically significant difference in both the LDFA and MPTA, with a mean difference of 0.32 ± 1.84 degrees and 0.84 ± 1.17 degrees respectively.

In terms of rotational alignment, post-operative measurements were averagely 1.63 ± 2.40 degrees away from the verified intra-operative plan. This difference was statistically significant. These are better illustrated in Table 2.

We were unable to reliably establish sagittal readings of the femoral component flexion and posterior tibial slope from the lateral knee radiographs taken post-operatively and did not proceed with any comparative analysis of these sagittal parameters.

### 3.4. Inter-Surgeon Comparisons

When comparing the post-operative measurements against the verified intra-operative plans, there were no statistically significant differences for LDFA and MPTA between the four surgeons however there was a difference for the HKA axis and rotational alignment (Table 3).

### 3.5. Hip-Knee-Ankle Axis

**Figure 3:**
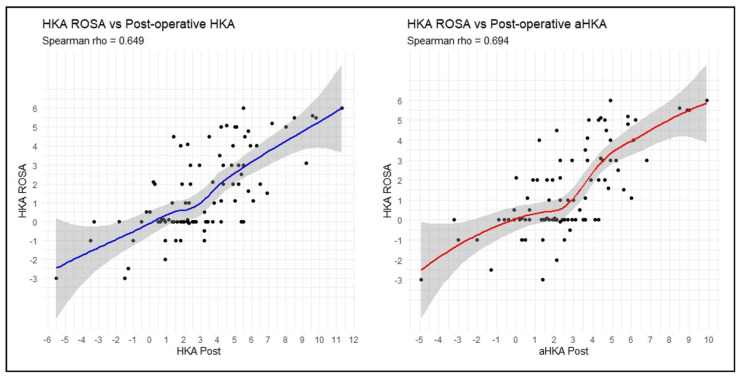
Correlation between the HKA captured on the ROSA Knee console (HKA ROSA) and post-operative alignment measurements. Left scatterplot shows correlation between HKA ROSA and post-operative measured HKA (HKA Post), demonstrating a moderately positive correlation (Spearman’s rho = 0.649). Right scatterplot depicts correlation between HKA ROSA and post-operative arithmetic HKA (aHKA Post), showing a higher moderately positive correlation (Spearman’s rho = 0.694). In both plots, a local polynomial (LOESS) regression curve (red or blue) illustrates the trend, and the shaded grey area represents the 95% confidence interval.

The verified intra-operative HKA axis captured by the robotic assistant had a moderately positive correlation with both the measured post-op HKA and the aHKA, with a marginally stronger correlation with the aHKA. These are better illustrated in Figure 3 below.

### 3.6. Intra- and Inter-Observer Reliability of Measurements

Intra- and inter-observer reliability was excellent for both LDFA and MPTA, with ICCs consistently above 0.90, confirming the reproducibility of radiographic measurements. The intra-observer coefficient for rotational alignment (ROT) was less consistent; however, the inter-observer scores range between 0.88 and 0.97, still representing good to excellent reproducibility. Table 4 captures this clearly.

### 3.7. Complications

There were no intra-operative or early post-operative complications, such as tracker pin site fractures or infections, wound dehiscence, surgical site infections, venous thromboembolic events, component overhang or notching.

## 4. Discussion

Comparison between intra-operative verified values and post-operative radiographic measurements demonstrated small but statistically significant deviations across all measured parameters. Nevertheless, the magnitude of these differences remained within clinically acceptable ranges [24], supporting the ROSA Knee System’s ability to reproducibly achieve planned alignment and implant positioning targets.

The results of our study show that the robotic system offers consistent reproduction of the intra-operatively planned HKA. Despite being statistically significant, the differences are within 1–2° of the intra-operative plan. With the rapid adoption and integration of robotic systems into the field of arthroplasty, maintaining an arbitrary target of neutral ± 3 degrees as per convention for manual TKA would negate one of the benefits of RATKA, which is the possibility of accommodating a wider range of HKA axes to obtain a well-balanced knee. The ability to accurately control the bone cuts and implant placement has led to the development and adoption of personalized alignment strategies [25,26]. The mean difference between post-op and intra-op planned HKA axis was 1.7 ± 2.1° and this mirrors that of Mancino and colleagues—who reported a discrepancy of 1.3 ± 1.3°—and Rossi et al., who published a mean difference of 1.2 ± 1.1°. Both authors utilized the ROSA Knee System in their studies [7,12]. The NAVIO^TM^ Surgical System (Smith & Nephew, Memphis, TN, USA) is another imageless robotic arthroplasty system; Bosco et al. recently published that the mean difference in their cohort of 101 patients was 1.2 ± 1.2° [27]. In our previous publication, we reported a mean difference of 0.8 ± 1.9° in that cohort of patients undergoing RATKA using the MAKO™ robotic system [5]. However, it must be pointed out a pre-operative CT scan is mandatory for patients undergoing surgery using the MAKO system [5].

In terms of implant positioning in the coronal plane, there was a statistically significant difference for both the LDFA and MPTA; however, the difference was only 0.3 ± 1.8° and 0.8 ± 1.2° respectively. Despite being statistically significant, these sub-degree discrepancies could be attributable to human error and may not actually translate to any noticeable clinical effect. Zhou et al. reported a difference of 1.8 ± 1.7° and 1.3 ± 1.1° for the LDFA and MPTA respectively in their retrospective analysis of 20 TKAs performed using the ROSA Knee System [28]. In a recently published systematic review, Zaidi et al. performed a pooled analysis on the included studies and reported an average discrepancy of 0.6 ± 0.1° for the LDFA and 0.4 ± 1.8° for MPTA [14]. These readings highlight the high degree of coronal accuracy and precision that the ROSA can offer.

Rotational accuracy is one parameter that has very limited data in the literature as this necessitates patients to undergo post-operative CT scans. In our cohort of patients, the mean difference in rotational positioning is 1.6 ± 2.4°, and this was statistically significant. We measured this against the trans-epicondylar axis (TEA) as it would not be possible to do so against the posterior-condylar axis (PCA). In their randomized controlled trial, Adamska reported a rotational accuracy of 1.3 ± 1.0° for the RATKA group using the CORI™ Robotic System (Smith & Nephew, Memphis, TN, USA) and 1.5 ± 1.1° for the RATKA group which utilized the NAVIO platform [23]. In our previous publication on the MAKO robot, we reported the rotational accuracy of that cohort of 155 patients to be 0.4 ± 1.5° [5]. Another point that warrants highlighting is the fact that even though these measurements in the axial plane are obtained from CT scans, there remains an ambiguity—as demonstrated by Lei and colleagues [29]. In their publication, six experienced arthroplasty surgeons were asked to identify the surgical transepicondylar axis (sTEA) from pre-operative CT scans. Each surgeon performed three measurements independently, with an interval of at least 15 days between each reading. Intra-surgeon reproducibility was moderate as adjudged by an intra-class correlation (ICC) coefficient of between 0.5 and 0.75. Inter-surgeon ICC was 0.528. The intra-class correlation coefficient for our study ranges between 0.78 and 0.93 whereas the inter-rater coefficient lies between 0.88 and 0.97. The increased variability concurs with the findings of Lei et al. and reaffirms the challenges in identifying the sTEA both pre-operatively and intra-operatively. However, we opine that the methodology outlined above remains the most accurate way of assessing post-operative rotational accuracy.

When taken as a whole, the degree of objectivity in accuracy and precision which the robot can consistently achieve should provide surgeons with confidence that their patients will be receiving the planned implant positioning and resultant gap balance, regardless of the alignment philosophy they adopt.

The HKA captured on the robotic console is done with the patient supine, thus eliminating the effects of weight-bearing. As shown by Paternostre et al., there exists a difference between weight-bearing and non-weight-bearing alignments which become more significant with increasing severity of arthritis [30]. This is attributable to the zone mechanical axis which represents the load-bearing axis in relation to the centre of the knee and the stretching of the convex-sided soft tissues. In our study, there is moderate correlation with both the measured post-op HKA, as well as the aHKA, with marginally higher correlation with aHKA. MacDessi and colleagues have extensively published on the aHKA being predictive of constitutional alignment [31]; with increasing emphasis placed on its restoration in attempts to further improve patient outcomes and satisfaction rates, surgeons who utilize ROSA Knee are now bolstered with further evidence and conviction that the HKA captured on screen is indeed representative of the aHKA.

Another benefit of RATKA would be the reduction in subjective intra-operative assessments, which accompanies all conventional TKAs. All robotic systems provide a certain degree of objectivity in terms of alignment, balancing and resultant HKA. Inter-surgeon consistency in coronal plane positioning supports the hypothesis that once proficiency is attained, surgical outcomes become less dependent on operator variability. However, there is a statistically significant difference in terms of HKA and rotational alignment. A plausible explanation would be that there exists a difference between standing and lying HKA axes, with standing HKA having the additional effects of weight-bearing and gravity. As shown in our results, the robot-captured HKA has moderate correlation with both the measured standing HKA and the aHKA, with a marginally higher correlation with aHKA. This slight difference could possibly be attributed to the effects of weight-bearing and difficulty of consistently identifying the sTEA as highlighted above.

This study is not without limitations. Firstly, this is a purely radiographic assessment with an observation of any potential early complications without consideration of clinical outcomes. The improved accuracy and precision of the robot as highlighted above may not necessarily translate to clinically meaningful improvement for the patients. Patient outcomes and long-term survivorship are multi-factorial. Regardless, our study provides data that the robotic assistant is indeed able to achieve good accuracy and precision with no increased risk of early complications. Next, we were unable to consistently ascertain the sagittal parameters of femoral component flexion and posterior tibial slope from the lateral knee radiographs, which would be the standard of care. A lateral long limb radiograph would allow this measurement to be performed, and we advocate for this extra radiographic view to be ordered for future studies to permit assessments in the sagittal plane and their impact on clinical outcomes. Additionally, despite our best efforts, the element of mild lower limb rotation and flexion cannot be excluded with absolute certainty, and this may have influenced the HKA readings. Subjecting patients to a standing long limb CT scan may improve accuracy of measurements however this would not be the standard of care on top of incurring additional radiation and financial costs. The exclusion of 34 patients who did not complete post-operative radiographic assessments could have introduced selection bias. However, recovery of these data was not possible, and all findings should be interpreted with recognition of the limitations stated above. Future studies with larger sample sizes and longer follow-up durations are warranted to further evaluate how these improvements in accuracy translate into clinical outcomes and to better elucidate the benefits of this robotic system.

## 5. Conclusions

The ROSA Knee System offers high accuracy and consistency in component placement and alignment restoration in TKA. Once the learning curve is surpassed, surgeons can expect reproducible results with minimal variation. Furthermore, the robot-captured HKA correlates well with both measured post-operative HKA and arithmetic HKA, supporting the use of ROSA in the adoption of various personalized alignment strategies.

## Figures and Tables

**Table 1:** Inclusion and exclusion criteria for enrollment into this study.

Inclusion
• Age > 18 years old
• End-stage bi- or tri-compartmental knee OA (Kellgren-Lawrence Grade 3 & 4) which have failed to respond to conservative treatment
• Undergoing Total Knee Arthroplasty (TKA) using the ROSA Knee System
Exclusion
• Inflammatory arthritis
• History of metabolic bone disease
• History of trauma or surgery in the affected knee
• History of infection in the affected knee
• Patients with only mono-compartmental knee osteoarthritis undergoing Partial Knee Arthroplasty (PKA)
• Revision TKA
• Conversion to TKA from previous PKA
• Severe knee deformities with resultant ligamentous laxity which requires implants of increased constraint
• Not willing to return for post-operative physiotherapy, follow-up assessments or post-op radiographic examinations

**Table 2:** Assessment of alignment and implant positioning comparing post-operative measurements against verified intra-operative values.

Parameter	Paired Differences				Significance, *p* Value ^α^
	Mean, Degrees (°)	Std. Deviation, Degrees (°)	95% Confidence Interval of Difference		
			Lower	Upper	
HKA	−1.66	2.09	−2.09	−1.23	<0.001
LDFA	−0.32	1.84	−0.69	0.06	0.034
MPTA	0.84	1.17	0.60	1.08	<0.001
Rotational	1.63	2.40	1.14	2.12	<0.001

^α^ Wilcoxon signed-rank test, *p* < 0.05 as significant at 95% CI. *HKA* Hip–Knee–Ankle axis; *LDFA* Lateral distal femoral angle; *MPTA* Medial proximal tibial angle.

**Table 3:** Comparison of post-operative measurements against verified intra-operative values for the various alignment parameters between surgeons.

Parameter	Mean ± SD, Degree (°)				Significance, *p* Value ^α^
	Surgeon A	Surgeon B	Surgeon C	Surgeon D	
**HKA**					0.007
Intra-op	0.8 ± 1.7	0.5 ± 1.0	3.4 ± 2.4	1.0 ± 1.4	
Post-op	2.8 ± 2.6	2.5 ± 1.6	4.0 ± 3.4	3.8 ± 4.4	
Difference	2.0 ± 2.0	2.0 ± 1.5	0.6 ± 1.9	2.9 ± 3.1	
**LDFA**					0.265
Intra-op	89.7 ± 1.6	89.4 ± 2.4	91.6 ± 2.0	90.5 ± 0.9	
Post-op	90.1 ± 1.5	90.5 ± 1.4	91.6 ± 2.5	90.4 ± 2.2	
Difference	0.3 ± 1.8	1.1 ± 2.1	0.0 ± 1.8	0.1 ± 1.5	
**MPTA**					0.604
Intra-op	88.5 ± 1.3	89.9 ± 1.0	88.5 ± 1.7	88.6 ± 1.3	
Post-op	87.7 ± 1.3	88.7 ± 1.2	87.8 ± 2.0	87.7 ± 1.5	
Difference	0.8 ± 1.1	1.2 ± 0.8	0.8 ± 1.5	1.0 ± 0.7	
**Rotational**					0.007
Intra-op	5.2 ± 3.7	2.1 ± 1.7	3.3 ± 2.6	2.8 ± 1.0	
Post-op	2.9 ± 2.1	2.3 ± 1.5	2.0 ± 1.2	1.6 ± 0.6	
Difference	2.3 ± 2.6	0.2 ± 1.0	1.3 ± 2.2	1.1 ± 1.3	

**^α^** Kruskal–Wallis test, *p* < 0.05 as significant at 95% CI. *HKA* Hip–Knee–Ankle axis; *LDFA* Lateral distal femoral angle; *MPTA* Medial proximal tibial angle.

**Table 4:** Assessment of intra- and inter-observer reliability of measurements.

Parameter	Rater	Intraclass Correlation Coefficient							
		Intra-Observer	95% Confidence Interval		Significance, *p*	Inter-Observer	95% Confidence Interval		Significance, *p*
			Lower Bound	Upper Bound			Lower Bound	Upper Bound	
LDFA	1	0.984	0.972	0.992	<0.001	0.991	0.982	0.995	<0.001
	2	0.993	0.986	0.996	<0.001				
	3	0.984	0.970	0.992	<0.001				
MPTA	1	0.980	0.963	0.990	<0.001	0.984	0.970	0.992	<0.001
	2	0.986	0.973	0.993	<0.001				
	3	0.978	0.959	0.989	<0.001				
ROT	1	0.934	0.884	0.966	<0.001	0.933	0.883	0.965	<0.001
	2	0.783	0.646	0.881	<0.001				
	3	0.929	0.875	0.963	<0.001				

Two-way mixed effects model where people effects are random, and measures effects are fixed, absolute agreement. *LDFA* lateral distal femoral angle; *MPTA* medial proximal tibial angle; *ROT* rotational alignment.

## Data Availability

The data that support the findings of this study are available on reasonable request from the corresponding author.

## Abbreviations

The following abbreviations are used in this manuscript:

aHKA	arithmetic Hip–Knee–Ankle axis
BMI	body mass index
CR	cruciate retaining
CT	computed tomography
HKA	Hip–Knee–Ankle axis
LDFA	lateral distal femoral angle
MPTA	medial proximal tibial angle
PKA	partial knee arthroplasty
PS	posterior stabilized
RATKA	robotic-assisted total knee arthroplasty
TEA	trans-epicondylar axis
TKA	total knee arthroplasty
sTEA	surgical trans-epicondylar axis
